# Transforming Nursing Practice to Optimize Care of Patients with Tuberculosis and Associated Comorbidities in the Kilimanjaro Region, Tanzania

**DOI:** 10.2174/0118744346356368241218080300

**Published:** 2025-01-31

**Authors:** Pendo Martha Joseph Shayo, Elyana A. Zewdie, Kenneth C. Byashalira, Nyasatu G. Chamba, Peter M. Mbelele, Ib C Bygbjerg, Troels Lillebaek, Dirk L Christensen, Scott K. Heysell, Stellah G. Mpagama

**Affiliations:** 1 Public Health and Disease Surveillance, Kibong’oto Infectious Diseases Hospital, Mae Street, Lomakaa Road, Siha Kilimanjaro 25401, Tanzania, United Republic of Tanzania; 2 Kilimanjaro Clinical Research Institute, Kilimanjaro Christian Medical University College, Moshi Kilimanjaro 25116, Tanzania; 3 Kilimanjaro Christian Medical University College, Faculty of Medicine, Moshi, United Republic of Tanzania; 4 Department of Internal Medicine, Kilimanjaro Christian Medical Center, Moshi, United Republic of Tanzania; 5 Nelson Mandela African Institute of Science and Technology, Arusha, Tanzania.; 6 Division of Infectious Diseases and International Health, University of Virginia, Charlottesville, VA 22908-1340, USA; 7 Pompano Lab, University of Virginia, Charlottesville, VA22908-1340, USA; 8 Global Health Section, Department of Public Health, University of Copenhagen, Copenhagen, Denmark; 9 International Reference Laboratory of Mycobacteriology, Statens Serum Institut, Copenhagen, Denmark

**Keywords:** Tuberculosis, Comorbidities, Nursing care, Patient-centered care, TB-DM, Multimorbidity

## Abstract

**Background:**

Managing tuberculosis (TB) patients with comorbidities requires a holistic and patient-centered approach. This study evaluated patient-centered care (PCC) experiences among TB patients with multimorbidity under the Adaptive Diseases Control Expert Program in Tanzania (ADEPT), with a focus on the TB/diabetes mellitus (DM) co-epidemic targeted by the program.

**Methods:**

A quantitative cross-sectional study was conducted involving TB patients with associated comorbidities receiving care from nurses trained in PCC through the ADEPT program. Patients were assessed for their interpretation of how they received PCC across eight dimensions of care.

**Results:**

All 39 participants with TB had at least one comorbidity (100%). The most common multi-morbidities were TB/HIV (59.0%) and TB/DM (12.8%). Evaluation of needs, values, and preferences revealed that participants strongly felt healthcare workers considered these aspects (mean score = 4.53; scale 1 minimum- strongly disagree to 5 maximum- strongly agree). Subsequent analysis showed varying evaluations across dimensions. Patients reported robust endorsement for receiving care that involved family and friends, with mean scores of 4.04, and for access to care, with a mean score of 4.40. However, physical comfort, coordination, continuity of care, education and emotional support received comparatively lower rankings.

**Conclusion:**

TB patients with multimorbidity under the ADEPT program experienced PCC. However, certain systemic issues such as physical spaces, coordination, transition of care, and information education did not meet patient’s needs in their own assessment, indicating opportunities for further quality improvement. Scaling up the ADEPT program within healthcare systems is likely to enhance care provision for TB patients with multimorbidity through a patient-centered approach.

## 1. INTRODUCTION

The sub-Saharan African region is confronted with the burden of emerging and re-emerging infectious diseases and increasing drug resistance. For instance, although it is an age-old disease, a record high number of people diagnosed with tuberculosis (TB) was registered in 2022 after COVID-related disruptions [1], including severe forms such as multidrug-resistant and extensively drug-resistant TB, a disease more common in people with malnutrition, human immunodeficiency virus (HIV), and among people with DM, as reported in recent studies [2, 3]. The management of these co-epidemics is intricate, given that patients with multimorbidities (coexistence of two or more chronic conditions) exhibit diverse needs and preferences, necessitating a comprehensive approach to care [4].

Comprehensive approaches to multimorbidity care aim to be patient-centered, yet the definitions of patient-centered care (PCC) continue to change in modern health systems. Schuster *et al*. [5] define PCC as follows: “providing care that demonstrates respect for and responsiveness to the individual patient’s preferences, requirements, and values, with an emphasis on ensuring that patient values serve as the guiding principles for all clinical decisions.” In a broader context, PCC encompasses a multitude of essential facets, including the prioritization of the patient’s needs and perspectives, the seamless integration and coordination of the patient’s care, the provision of comprehensive multidisciplinary care, ensuring the availability of easily accessible supportive information systems, and the establishment of continuous learning systems [6]. PCC recognizes that healthcare is not just about treating diseases but also about supporting patients as whole individuals with unique needs and preferences [7]. Nursing roles, as outlined in the World Health Organization (WHO) End TB Strategy, underscore the responsibility of nurses to uphold PCC consistently throughout the treatment process, from the initial interaction to the culmination of treatment and transitioning to post-TB rehabilitation [8]. This objective can be achieved by empowering nurses with the necessary tools and knowledge.

A report from Tanzania, based on information gathered through a focus group discussion with patients, highlighted that nurses need to be at the forefront of delivering best practices in the care of TB patients [9]. Despite the existence of multiple continuous professional development (CPD) programs focusing on TB and its severe forms within Tanzania, a noticeable gap persists. We hypothesize that empowering nurses in PCC practice through incremental training can enhance their skills to effectively implement the End TB Strategy, manage emerging infectious diseases and address multimorbidity. Empowerment means gaining confidence and the ability to successfully face challenges, whether as an individual or within a group [10]. In this context, empowered nurses are those who have gained from the mentorship process and are now equipped to support and mentor their colleagues.

Our initiative trained graduate nurses from pilot regions (Dar es Salaam, Iringa, and Kilimanjaro) to provide comprehensive care for TB patients, the majority of whom would be expected to have multimorbidity [HIV, DM, malnutrition]. This training was facilitated by the ADEPT model [11]. The training involved web-based learning, classroom discussions, and clinical attachments, fostering practical application through the Gibbs reflection cycle [11]. Empowered nurses not only raised their practice standards but also served as mentors for colleagues in nearby healthcare facilities. Subsequently, we conducted an assessment of a cohort of patients with multiple morbidities who had been attended to by mentored nurses. The objective was to ascertain the extent to which the care provided was aligned with a patient-centered approach, taking into account individual needs and preferences.

## 2. METHODS

### 2.1. Study Design and Population

A cross-sectional study was conducted to evaluate PCC in health facilities that implemented the ADEPT model. The ADEPT model aims to implement international guidelines for TB and associated comorbidities, focusing on the early diagnosis and personalized treatment of patients with TB and comorbidities, using TB-DM as a case study [11–13].

This study employed a quantitative approach and gathered data from various healthcare facilities located within the Kilimanjaro region of Tanzania. Specifically, the selected facilities were strategically chosen from the three districts involved in the ADEPT study: Same, Siha, and Moshi Municipal. These facilities, including Mawenzi Regional Referral Hospital (RRH) and St. Joseph District Designated Hospital (DDH) in urban Moshi, Same District Council Hospital in suburban Same, Majengo Health Center and Pasua Health Center in urban Moshi, and Siha Health Center in rural Siha District, were all integral parts of the ADEPT study. As a subsection of the ADEPT study, this research focuses on assessing PCC within the Kilimanjaro region, leveraging facilities already involved in the broader initiative. The study was guided by eight principles of PCC identified by Picker Institute and Harvard Medical Schools as described in section 2.7.

### 2.2. The Inclusion Criteria

1. Patients eligible for inclusion in the study were those diagnosed with TB and undergoing treatment, with the presence of at least one additional co-morbidity. Co-morbidities considered in this study included but were not limited to HIV/AIDS, DM, malnutrition, hypertension, kidney failure, liver cirrhosis, peptic ulcer disease (PUD), hypotension, renal disease, silicosis, or cancer of the lungs.
2. Patients eligible for inclusion were those undergoing TB treatment for at least three days or who had completed treatment within the past three months. This range allowed us to capture a variety of experiences, though we acknowledge that feedback may vary depending on the length of time under care.
3. Patients had to be able to attend the facility for interviews. Efforts were made to contact all eligible patients before their facility visit, and those who were reachable by phone were invited to the facility. Transportation costs were reimbursed for patients who attended in person. Patients who were unable to attend the facility were offered alternative interview arrangements. This approach aimed to accommodate diverse patient circumstances and promote inclusivity in the study.

### 2.3. Recruitment of Study Participants

The recruitment strategy for this quantitative study employed a pragmatic approach to ensure comprehensive representation. The Kilimanjaro region was purposively selected due to its proximity to Kibong’oto Infectious Diseases Hospital, which played a leading role in the ADEPT mentorship and training program. This proximity facilitated easier provision of consultancy and assessment in these sites. Additionally, the Kilimanjaro region was chosen because of its relatively higher prevalence of DM compared to other regions [13, 14]. All eligible participants were given an equal chance of being involved, and detailed information about the study was provided to potential candidates before obtaining consent. It is important to note that this specific study exclusively focused on the experiences of TB patients managing multimorbidity in the Kilimanjaro region due to these strategic reasons.

### 2.4. Ethical Approval

The study was approved by the Tanzanian National Institute of Medical Research as part of the ADEPT protocol. Following approval, additional permission to proceed was obtained from regional medical authorities and facility charges.

### 2.5. Data Collection

The study involved conducting structured interviews with 39 clients who had both TB and comorbidities (multimorbidity). Interviews were conducted using a carefully designed questionnaire.

### 2.6. Sample Size

The sample size of 39 participants was not determined through formal calculations but was instead drawn from the entire eligible population within the study’s catchment area in the Kilimanjaro region. This strategy aimed to empower the nursing cadre and thoroughly explore the unique context within the study’s specific focus. Notably, efforts were made to include patients who did not physically visit the facilities during data collection, ensuring a comprehensive representation of perspectives. All eligible participants were contacted through phone calls, ensuring a representative sample and providing valuable insights within the Kilimanjaro region.

Furthermore, our methodological approach aligns with findings from other studies conducted in similar contexts. For example, “Views of patients with multi-morbidity on what is important for patient-centered care in the primary care setting” and “I am not just a place for implementation. I should be a partner”: a qualitative study of patient-centered care from the perspective of diabetic patients in Saudi Arabia,” both demonstrated the utility of qualitative research with relatively small sample sizes in elucidating patient perspectives and care delivery practices [15, 16]. Similarly, our study, despite its modest sample size, contributes meaningful insights into TB management and patient-centered care practices in the Kilimanjaro region.

Additionally, we referenced a study conducted along the Myanmar–Thailand border, which investigated nutritional intake among patients undergoing TB treatment, to provide context and support for our findings [17]. This study further underscores the relevance and adequacy of our sample size in enhancing the understanding of TB management and patient-centered care practices, though it acknowledges that the sample size may be subject to further scrutiny.

### 2.7. Data Collection Tools

For the assessment of PCC among patients with multimorbidity in the primary care setting, we utilized the Patient-Centered Primary Care (PCPC) instrument, as described by Cramm and Nieboer [18]. The validated version retained the original eight dimensions of PCC but expanded to include 36 items for comprehensive assessment, as reported in Cramm and Nieboer’s work [18]. These dimensions include respect for patients’ preferences, access to care, emotional support, information and education, involvement of family and friends, continuity and secure transition between healthcare settings, physical comfort, and coordination of care.

Given that the questionnaire was originally in English, it was later translated into Swahili. Before actual data collection, the three data collectors were given training on how to use the questionnaire during fieldwork activities. No discrepancies in data collection were noted within the training activities.

### 2.8. Data Management and Analysis

Data were gathered using paper forms, which were then entered and cleaned using the Statistical Package for Social Science (SPSS) version 25. The findings were analyzed and summarized using frequencies and percentages and presented in a tabular format.

## 3. RESULTS

The results of the study are presented in this section. Table 1 provides a summary of the demographic characteristics of the participants, followed by the main findings.

The assessment involved a five-point Likert scale, where respondents could indicate their level of agreement or disagreement on a range consisting of “Strongly agree” to “Strongly disagree”. This scale is considered an interval scale; therefore, the mean holds great significance in determining overall perception. Specifically, scores between 1 to 1.8 indicate a strong disagreement, scores between 1.81 to 2.60 represent a somewhat disagreement, scores between 2.61 to 3.40 signal a neutral standpoint, scores between 3.41 to 4.20 suggest a somewhat agreement, and scores between 4.21 to 5 imply a strong agreement. The themes assessed for PCC encompassed eight dimensions. These dimensions included patient preferences, physical comfort, coordination of care, continuity and transition, involvement of family and friends, emotional support, access to care, and information and education. The findings for each dimension are summarized in the following tables.

Firstly, patient preferences regarding treatment decision-making and support were assessed (Table 2). These preferences were assessed through a series of questions aimed at understanding the level of involvement and support perceived by patients in their treatment journey.

The mean upper limit of 4.82 suggests a general consensus among participants regarding the statements. However, it’s important to note the variability in responses, especially for items with wider standard deviations. For example, the item “If the influence of treatment on my life was taken into account” had a mean score of 4.15, accompanied by a relatively high standard deviation of 1.25. This indicates that responses varied widely. Additionally, we calculated the overall mean and standard deviation for all questions in the category/table to be 4.53 and 0.28, respectively. This indicates an overall positive perception, with data points closely concentrated around the mean. It underscores a strong consensus among participants regarding the attentive consideration by nurses of patients’ needs, values, and preferences.

Secondly, we investigated participants’ perceptions of physical comfort within healthcare settings, as documented in Table 3.

Mean scores across various dimensions, including attention to the patient, cleanliness, and privacy maintenance, indicate generally low levels of perceived comfort. For instance, the mean scores range from 1.26 to 1.74, with an overall mean of 1.54 and a standard deviation of 0.31, suggesting a need for improvement in these areas. Furthermore, the standard deviations highlight the variability in participant responses, indicating diverse perspectives among respondents. For example, while the mean score for attention to the patient is 1.64, the standard deviation of 1.06 suggests significant variability in how participants perceive this aspect. This variability underscores the importance of considering individual experiences when addressing issues related to physical comfort. Overall, these findings emphasize the importance of targeted interventions aimed at enhancing physical comfort in healthcare settings.

Thirdly, we investigated the coordination of care within healthcare settings, as shown in Table 4.

In our analysis, the overall mean and standard deviation for all aspects related to coordination of care are presented in Table 4. The mean scores for clarity of communication among the medical team, consistency of care provided by practitioners, patients’ awareness of the care coordinator, and accessibility for questions ranged from 1.41 to 1.80. The overall mean across these dimensions was approximately 1.57, with a standard deviation of approximately 0.20, indicating relatively low levels of perceived coordination of care. However, it’s important to note the variability in responses, as reflected by the standard deviations. For instance, while the mean score for clarity of communication was 1.41, the standard deviation of 0.82 suggests diverse perceptions among participants. This variability underscores the range of experiences regarding the coordination of care among our respondents.

Fourthly, we investigated participants’ perceptions of continuity and transition of care, as shown in Table 5.

After analyzing the data presented in Table 5 regarding continuity and transition aspects, it’s evident that participants’ responses varied across different variables, as indicated by mean scores ranging from 1.62 to 1.73. To offer a comprehensive perspective, we computed the overall mean and standard deviation for all questions in this category. The resulting overall mean of approximately 1.66 suggests a moderate level of agreement, on average, among participants regarding these aspects. Furthermore, the overall standard deviation of around 0.09 implies that responses were relatively consistent around this mean value, indicating a narrow dispersion of data points. These findings highlight both the general level of agreement and the consistency of responses across the surveyed variables.

Fifthly, participants’ perceptions of emotional support were assessed, as depicted in Table 6.

The analysis of Table 6 reveals a moderate level of emotional support provided to patients across the surveyed variables. Mean scores ranged from 1.90 to 2.82, indicating varying levels of agreement among participants regarding the provision of emotional support. The overall mean of approximately 2.20 suggests a moderate level of agreement. However, the standard deviations ranged from 1.23 to 1.55, indicating variability in responses. This variability suggests differences in the level of support reported by individual participants, emphasizing the need for consistent and comprehensive emotional support in healthcare settings.

Sixthly, participant’s access to care was assessed, as illustrated in Table 7.

After analyzing the data presented in Table 7 concerning access to care, the mean scores for various aspects ranged from 3.80 to 4.54. These scores indicate a generally positive perception among participants regarding the accessibility and availability of healthcare services. The overall mean score, calculated across all variables, is approximately 4.04, with a standard deviation of approximately 0.28. This suggests a relatively narrow spread of data points around the mean, indicating a degree of consistency in participants’ responses. These findings underscore the importance of ensuring accessible healthcare services for patients. While the overall perception is positive, there may still be areas for improvement to ensure equitable access and reduce disparities in healthcare delivery.

Seventhly, we evaluated the information and education aspects of care, as depicted in Table 8.

After examining the data presented in Table 8, it is apparent that participants have varying levels of agreement regarding the information and education aspects, as reflected in the mean scores, which range from 1.54 to 2.08. The overall mean score across all variables is approximately 1.78, with a standard deviation of approximately 0.19. This suggests a relatively narrow spread of data points around the mean, indicating a degree of consistency in participants’ responses. These findings highlight the importance of ensuring effective communication and access to relevant information for patients, as well as the need for consistent educational resources in healthcare settings.

Lastly, participants’ perceptions regarding the involvement of family and friends were assessed as described in Table 9.

After analyzing the data presented in Table 9, focusing on the involvement of family and friends in healthcare decisions, it is apparent that participants generally perceive a high level of involvement from their relatives. The mean scores for various aspects ranged from 4.24 to 4.59, indicating a strong agreement among participants regarding the active participation of family members in the treatment process. Moreover, the overall mean score, calculated across all variables, is approximately 4.40, with a standard deviation of approximately 0.04. This suggests a narrow spread of data points around the mean, reinforcing the high level of consensus among participants regarding the involvement of family and friends in healthcare decisions. These findings underscore the significant role that family members play in the healthcare journey of patients. The high mean score and low standard deviation imply widespread agreement among participants, highlighting the importance of involving family members in healthcare decisions.

## 4. DISCUSSION

Overall, the study showed that TB patients with comorbidities, when served by nurses trained by the ADEPT methodology, had positive experiences with patient-centered care as estimated by 8 dimensions of the PCC tool.

First, the finding that most participants strongly agreed that their needs, values, and preferences were considered by healthcare workers aligns with the most consistent tenet of PCC. However, it is important to note that some participants did not have the same positive experience. Therefore, it is necessary to perform further research to understand why some patients felt that their needs and preferences were considered, whereas some felt otherwise. Importantly, involvement in decision-making from a WHO standard [19] has been proven to improve treatment outcomes [20]. Other similar studies to ours that explored the needs and preferences of TB patients, found that patients preferred a shorter duration of TB treatment [21] and greater privacy [22]. Yet in many settings, these patient preferences are not prioritized, as an example reported by Mkopi *et al*. [23] where patients with TB routinely did not have their preferences considered in their treatment plan. Our findings suggest that patients in the ADEPT program felt their needs were considered by their providers. However, Further investigation is needed to assess patients’ willingness to engage in shared decision-making based on their involvement level.

Patients’ perceptions of physical comfort, such as attention to their feelings and complaints, cleanliness of the waiting venue, comfort of the waiting area, and privacy maintenance, were relatively low, highlighting areas that need attention. There is a need for interventions aimed at improving the physical environment and ensuring patient comfort during healthcare visits to prevent the negative consequences that could result from these experiences. This aligns with the findings of a systematic review by Cazabon *et al*., which investigated user experiences and patient satisfaction in TB care across low- and middle-income countries. Cazabon *et al*. reported a notable association between patient dissatisfaction with TB care services and an increased likelihood of being lost to follow-up. Therefore, we reference the increased risk of patients terminating treatment before the end.

Given the low scores for cleanliness and comfort in the waiting areas, it is essential to implement targeted interventions. These should include establishing regular cleaning schedules, ensuring comfortable seating arrangements, and improving ventilation. Upgrading hospital infrastructure to create more comfortable waiting areas would not only enhance patient experience but also support the operationalization of infection prevention measures, a persistent challenge in low- and middle-income countries like Tanzania [24].

Engaging patients in the design of their care environment is also crucial, as the benefits of such involvement outweigh the challenges of the process. A study by Annemans *et al*. demonstrated that incorporating patient experience data into the design process can significantly enhance the functionality and comfort of healthcare settings. In workshops where patient experience was used to inform design decisions, the resulting environments were found to be more attuned to patient needs, leading to improved satisfaction and better overall patient outcomes [25]. Additionally, training programs for healthcare staff can raise awareness about the importance of physical comfort and provide strategies for ongoing improvement.

While it is encouraging that patients from many lower-level facilities reported satisfactory levels of cleanliness and waiting area comfort, it is notable that patients from Same DC expressed lower satisfaction in these areas compared to those from higher-level facilities like Mawenzi. This discrepancy may be influenced by differences in facility capacity and resources. Understanding these variations is crucial for devising tailored interventions to promote equitable healthcare experiences across different facilities.

The coordination of care is another area that receives lower ratings. Participants reported that communication from the medical team and consistency of care provided by practitioners were not optimal. These findings are similar to those of a qualitative study conducted in Cape Town, South Africa, which explored socioeconomic and health system-related factors as contributors [26]. This highlights a potential gap in the healthcare system that needs to be addressed to ensure seamless coordination and effective continuity of care for TB patients. Efforts should be made to enhance communication among healthcare teams and improve consistency in care delivery. The recommendation is to invest in empathic communication to make patients feel seen and respected and to enhance the development of trust [27, 28]. The incorporation of technological advancements such as mHealth holds promise in addressing these challenges. As demonstrated by studies conducted in such diverse settings as Irkutsk, Siberia, mHealth interventions have proven effective not only in optimizing treatment but also in enhancing communication between providers and patients [29, 30]. By adopting mHealth strategies, we can bridge communication gaps within healthcare teams, improving overall coordination and integration of services. This, in turn, can boost patient confidence in the healthcare system and lead to better treatment outcomes. Importantly, efforts should be made to address potential barriers to mHealth adoption, as noted in a study conducted by Zakerabasali *et al*. [31].

Furthermore, findings related to information and education revealed that patients felt less informed, with the information provided not always being well explained. This contrasts with Chamba et al’s. study on dual TB and DM management in Tanzania, which was also part of the ADEPT program and focused on enhancing patient care [14]. Although the current study found that patients’ preferences were considered in decision-making, indicating some level of involvement, the perceived lack of comprehensive information suggests that further efforts are needed to improve communication. Tailored strategies should be implemented to ensure patients are fully informed and empowered, ultimately bridging existing information gaps.

The positive ratings for the involvement of family and friends indicate that patients felt supported and recognized the role that their loved ones played in their treatment process. Healthcare providers should continue to involve family members and provide them with the appropriate information and support. Additionally, it is important to address the questions and concerns of family members and ensure that they are part of the decision-making process with the consent of the patient. The study in Haydom, Tanzania, shed light on the severe strain TB places on families. Consequently, recommendations underscore the importance of holistic approaches to mitigate TB’s impact. This entails providing social, emotional, and economic support to alleviate the burdens faced by patients, caregivers, and siblings [32]. Similarly, the study conducted by Duarte *et al*. highlighted the importance of social interventions that require collaborative efforts in managing patients with multi- morbidities [33].

This study is not without limitations. The sample size, determined by including all eligible participants, may be considered small, limiting the generalizability of the study’s findings due to the relatively few participants in these specific health categories. Additionally, the study relies on self-reported measures, introducing potential biases. Future research should address these limitations by including qualitative studies on the lived experiences of patients, their families, and healthcare workers to provide deeper insights into the impact of these conditions.

## CONCLUSION

While the study identified both strengths and areas needing improvement in PCC for TB patients with comorbidities, patient preferences emerged as a crucial indicator of their perception of PCC, receiving high scores. This underscores the importance of patient preferences in assessing care quality. Future research should further explore how these preferences can be integrated into PCC evaluations to enhance understanding and improve care. Similarly, categories such as access to care and involvement of family and friends also performed well. However, specific aspects such as physical comfort, coordination of care, continuity and transition, emotional support, and information and education did not receive as high ratings, indicating areas for enhancement. As proven by other researchers in their review that aimed to explore the implementation of patient-centered interventions [34], addressing these specific aspects will be crucial in further enhancing the patient experience and treatment outcomes for TB patients.

## Figures and Tables

**Table 1.** Characteristics of the participants served by selected health facilities in kilimanjaro region of Tanzania.

Characteristic		N	%
		39	100
Health Facilities	Majengo Health center	3	7.7
	Mawenzi Regional Referral Hospital	17	43.6
	Pasua Health center	4	10.3
	Same District Council Hospital	3	7.7
	Siha Health center	4	10.3
	St Joseph District Designated Hospital	4	20.5
-	-	-	-
Sex	Female	19	48.7
	Male	19	48.7
	Unidentified (did not disclose)	1	2.6
	-	-	-
Age (years)	20–29	3	7.7
	30–39	11	28.2
	40–49	13	33.3
	50–59	8	20.5
	60–69	2	5.1
	70–79	1	2.6
	80 or above	1	2.6
	-	-	-
Education level	-	-	-
	Completed Form Four	3	7.7
	Form One to Form Three	3	7.7
	Primary Education	29	74.4
	Not completed primary education	4	10.2
Duration of treatment	<3 month	11	28.2
	3–6	19	49
	TB Treatment Completion (Past 3 Months)	7	17.9
Diagnosis	-	-	-
	TB/DM	5	12.8
	TB/DM/HIV	1	2.6
	TB/DM/Silcosis	1	2.6
	TB/HIV	23	59.0
	TB/HIV/Malnutrition	2	5.1
	TB/Hypertension	1	2.6
	TB/Kidney failure	1	2.6
	TB/Liver cirrhosis	1	2.6
	TB/Malnutrition	1	2.6
	TB/Peptic ulcer disease	1	2.6
	TB/Renal disease	1	2.6
	TB/SilIcosis/Cancer of the lungs	1	2.6
**Total**	**39**	**100.0**	

**Table 2.** Patient preferences.

-	N	Minimum	Maximum	Mean	Std. Deviation
If the patient was taken seriously	39	3.00	5.00	4.82	.45
If patient preferences on treatment were considered	39	1.00	5.00	4.46	1.05
If the patient was involved in making a decision on treatment	39	1.00	5.00	4.44	1.19
If the influence of treatment on my life was taken into account	39	1.00	5.00	4.15	1.25
If the patient was helped to determine treatment goals	38	3.00	5.00	4.63	.63
If the patient felt supported to achieve his treatment goals	39	3.00	5.00	4.56	.68
If advice that patient received was useful	38	1.00	5.00	4.63	.82
**Overall**	-	-	-	**4.53**	**0.28**

**Table 3.** Physical comfort.

-	N	Minimum	Maximum	Mean	Std. Deviation
If attention was given to the patient	39	1.00	5.00	1.64	1.06
If attention was given to patient’s feelings and complaints	39	1.00	5.00	1.74	1.04
If the waiting venue was clean	39	1.00	3.00	1.26	.50
If the waiting venue was comfortable	39	1.00	3.00	1.36	.58
If the privacy of patient was maintained	39	1.00	5.00	1.69	1.32
**Overall**	-	-	-	**1.54**	**0.31**

**Table 4.** Coordination of care.

-	N	Minimum	Maximum	Mean	Std. Deviation
If communication of the medical team was clear	39	1.00	5.00	1.41	.82
If consistent care is provided by practitioners	39	1.00	5.00	1.46	.85
If the patients know the one coordinating the care	39	1.00	5.00	1.80	1.32
If the patient could easily contact someone with questions	39	1.00	5.00	1.62	1.09
**Overall**	-	-	-	**1.57**	**0.20**.

**Table 5.** Continuity and transition.

-	N	Minimum	Maximum	Mean	Std. Deviation
If patient was informed well communicated, any movement	37	1.00	5.00	1.73	.96
If the patient was informed during handling over	39	1.00	5.00	1.67	1.16
If health advices were well attuned to each other	39	1.00	5.00	1.64	1.20
If information provided at TB clinic was in line with treatment of other care	39	1.00	5.00	1.62	1.09
**Overall**	-	-	-	**1.66**	**0.09**

**Table 6.** Emotional support.

-	N	Minimum	Maximum	Mean	Std. Deviation
If emotional support was provided to the pt	39	1.00	5.00	2.03	1.35
If attention was given to signs of emotional discomfort	38	1.00	5.00	1.90	1.23
If patient was informed about the possibilities of more intensive emotional	39	1.00	5.00	2.82	1.55
If attention was paid to the health impact on pts life	39	1.00	5.00	2.08	1.38
**Overall**	-	-	-	**2.20**	**0.12**

**Table 7.** Access to care.

-	N	Minimum	Maximum	Mean	Std. Deviation
If patient can reach to health facility without any difficulties	39	1.00	5.00	3.80	1.63
If health services were easily accessible	37	1.00	5.00	4.05	1.47
If the patient could easily schedule an appointment	39	1.00	5.00	3.97	1.35
If patient’s waiting time was not long 1	39	2.00	5.00	4.54	.82
If the patient was able to request a repeated recipe	39	1.00	5.00	3.82	1.17
**Overall**	-	-	-	**4.04**	**0.28**

**Table 8.** Information and education.

-	N	Minimum	Maximum	Mean	Std. Deviation
If the patient was well informed	39	1.00	5.00	1.77	1.16
If the patient received information that are well explained	39	1.00	5.00	1.74	1.09
If the patient was able to access easily own data	39	1.00	5.00	2.08	1.48
If the patient could ask all the questions, she/he wants	39	1.00	5.00	1.54	.97
**Overall**	-	-	-	**1.78**	**0.19**.

**Table 9.** Involvement of family and friends.

-	N	Minimum	Maximum	Mean	Std. Deviation
If a relative is involved in treatment after patient consented	39	1.00	5.00	4.59	.97
If attention was given to care and support provided by family members	37	2.00	5.00	4.38	.95
If attention was given to family members for possible questions	38	2.00	5.00	4.24	1.05
**Overall**	-	-	-	**4.40**	**0.04**

## Data Availability

The data supporting the findings of this article are not stored in a public repository but can be made available by the corresponding author upon reasonable request from corresponding author [P.S.].

## LIST OF ABBREVIATIONS

PCC: patient-centered care
RRH: Regional Referral Hospital
DDH: District Designated Hospital
PUD: peptic ulcer disease
PCPC: Patient-Centered Primary Care
SPSS: Statistical Package for Social Science
